# Mediastinal Tumor in a Boy With GnRH-Independent Precocious Puberty and Fluctuating β-HCG Levels

**DOI:** 10.1210/jcemcr/luae169

**Published:** 2024-09-27

**Authors:** Smadar Shilo, Shirah Amar, Noa Shefer Averbuch, Efraim Rosenbaum, Moshe Phillip, Liora Lazar

**Affiliations:** The Jesse Z and Sara Lea Shafer Institute for Endocrinology and Diabetes, National Center for Childhood Diabetes, Schneider Children's Medical Center of Israel, Petah Tikva 4920235, Israel; Faculty of Medical and Health Sciences, Tel Aviv University, Tel Aviv-Yafo 6997801, Israel; Department of Hematology-Oncology, Schneider Children's Medical Center of Israel, Petah Tikva 4920235, Israel; The Jesse Z and Sara Lea Shafer Institute for Endocrinology and Diabetes, National Center for Childhood Diabetes, Schneider Children's Medical Center of Israel, Petah Tikva 4920235, Israel; Faculty of Medical and Health Sciences, Tel Aviv University, Tel Aviv-Yafo 6997801, Israel; The Raphael Recanati Genetics Institute, Rabin Medical Center, Beilinson Campus, Petah Tikva 4941492, Israel; Leumit Health Services, Beit Shemesh 9909003, Israel; The Jesse Z and Sara Lea Shafer Institute for Endocrinology and Diabetes, National Center for Childhood Diabetes, Schneider Children's Medical Center of Israel, Petah Tikva 4920235, Israel; Faculty of Medical and Health Sciences, Tel Aviv University, Tel Aviv-Yafo 6997801, Israel; The Jesse Z and Sara Lea Shafer Institute for Endocrinology and Diabetes, National Center for Childhood Diabetes, Schneider Children's Medical Center of Israel, Petah Tikva 4920235, Israel; Faculty of Medical and Health Sciences, Tel Aviv University, Tel Aviv-Yafo 6997801, Israel

**Keywords:** Gonadotrophin-independent puberty, hCG test, Klinefelter syndrome

## Abstract

Gonadotropin-releasing hormone (GnRH(-independent premature puberty in boys, characterized by elevated β-human chorionic gonadotropin (β-hCG) levels, can indicate a secreting germ cell tumor (GCT). These tumors are rare but more common in individuals with Klinefelter syndrome (KS). We present a case of a 7.3-year-old boy with precocious puberty. Physical examination revealed bilateral testicular volumes of 8 to 10 mL and Tanner stage 3 secondary sexual characteristics (genitalia G3, pubic hair P3). His skeletal age was 12 years. Biochemical tests showed suppressed gonadotropin levels, elevated testosterone, and increased β-hCG of 86.6 mIU/mL (86.6 IU/L, reference range: <5 mIU/mL, <5 IU/L). Imaging, including magnetic resonance imaging (MRI), chest x-ray, whole-body computed tomography (CT), and testicular ultrasound, were interpreted as normal except for a small pineal cyst. Karyotype testing confirmed KS. Over 10 months, β-hCG levels fluctuated between 1 to 105 mIU/mL (1-105 IU/L). When β-hCG was 3.6 mIU/mL (3.6 IU/L), a fluorodeoxyglucose positron emission tomography–CT (FDG PET-CT) scan revealed a mediastinal tumor. The tumor was surgically removed and identified as a mature teratoma. This case underscores the importance of karyotype testing and repeated imaging in boys with premature puberty and elevated β-hCG levels, even if β-hCG levels decrease spontaneously and remain low.

## Introduction

Precocious puberty (PP) is defined as the development of secondary sexual characteristics in girls before age 8 years and in boys before age 9 years (which corresponds to 2 to 2.5 SDs below the mean age of puberty onset in the general population) [1]. PP is classified into 2 types: gonadotropin-releasing hormone (GnRH)-dependent/central precocious puberty (CPP) and GnRH-independent PP (GIPP). While CPP commonly reflects idiopathic early maturation of the hypothalamic-pituitary-gonadal axis, GIPP is typically the first clinical sign of underlying disease [2]. GIPP in boys may be caused by Leydig cell tumors, β-human chorionic gonadotropin (β-hCG)-secreting tumors, congenital adrenal hyperplasia, familial male-limited precocious puberty, McCune-Albright syndrome, aromatase excess syndrome, and exogenous sex steroids or endocrine-disrupting chemicals [2]. Germ cell tumors (GCTs) account for approximately 3% of all malignant tumors in children [3]. However, these tumors are substantially more common in Klinefelter syndrome (KS), with several reports showing an incidence of 1.5 per 1000 in patients with KS and an odds ratio (OR) of 50 compared to the healthy population for extragonadal GCT. Notably, most extragonadal GCT in children with KS are mediastinal or in the central nervous system [4]. These tumors may manifest as precocious puberty in boys since β-hCG activates luteinizing hormone (LH) receptors on the Leydig cells, resulting in increased testosterone production [5].

Here, we report a case of a challenging diagnosis of a mediastinal GCT in a 7.3-year-old boy who presented with GIPP, fluctuating serum β-hCG levels, and initial imaging scans interpreted as normal. Following his presentation, karyotype testing confirmed the diagnosis of KS. After 10 months of follow-up, he was diagnosed with a mediastinal GCT detected on a fluorodeoxyglucose positron emission tomography–computed tomography (FDG PET-CT) scan.

## Case Presentation

In February 2022 a previously healthy 7.3-year-old boy was referred to the endocrinology clinic due to signs of sexual precocity. Several weeks earlier, his mother noticed the development of a deep voice and the appearance of pubic hair. His past medical history was unremarkable. He was on no prescribed medications and denied using any other drugs or supplements. His development was reported to be in the normal range, although he reached developmental milestones later than his siblings. Beginning at age 3, he had a tendency to chew nonfood substances, such as pencils and erasers.

He was born after an uneventful pregnancy to healthy, nonconsanguineously married parents. He had 8 healthy siblings with normal growth and development. Mid-parental height was 182.5 cm and there was no family history of precocious puberty.

On admission, the boy's height was 143.0 cm (SDS +3.32), and weight—38.5 kg (SDS +2.36). His skeletal age was 12 years. General physical examination was normal except for the adult physique. The pubertal staging (according to Tanner) was 3 for genital development and pubic hair and 1 for axillary hair (G3, P3, A1). The testicular volume (TV) of both testes was 8 to 10 mL with no palpable mass. A stretched penile length was 11 cm (normal range for age 6.2 ± 1 cm). No gynecomastia was palpated and no skin lesions suggestive of McCune-Albright syndrome were noticed.

## Diagnostic Assessment

Table 1 displays the laboratory work-up findings. At the time of presentation, testosterone levels were 24.81 nmol/L (715.7 ng/dL) (normal prepubertal range <0.24-0.9 nmol/L, <6.92-25.96 ng/dL), with suppressed basal and only slightly increased post–luteinizing hormone releasing hormone (LHRH) stimulation LH and follicle-stimulating hormone (FSH) levels. Basal androgen levels, including 17-hydroxyprogesterone (17OHP), dehydroepiandrosterone sulfate (DHEA-S), and androstenedione, were within the normal prepubertal range, and 17OHP and cortisol responded normally to adrenocorticotropin (ACTH) stimulation. β-hCG levels were high, 105 mIU/mL (105 IU/L, normal range <5 mIU/mL, 5 IU/L), whereas α-fetoprotein levels were within the normal range, at 0.6 IU/mL (72.6 ng/dL) (normal range <5.8 IU/mL, 701.8 ng/dL). The results of general laboratory testing, including a complete blood count, biochemistry, thyroid functions, and inflammatory markers, were all normal.

**Table 1.** Results of laboratory investigations

Investigation	Result	Normal range
Initial presentation		
LH	<0.05 IU/L (<0.05 mIU/mL)	<5 IU/L (<5 mIU/mL) (prepubertal)
FSH	<0.09 IU/L (<0.09 mIU/mL)	0-2.8 IU/L (0-2.8 mIU/mL) (prepubertal)
Testosterone	715.7 ng/dL (24.81 nmol/L)	<6.9-25.96 ng/dL (<0.24-0.9 nmol/L) (prepubertal)
Estradiol	103 pmol/L (28.1 pg/mL)	<40 pmol/L (<10.9 pg/mL) (prepubertal)
Progesterone	<0.3 nmol/L (<0.094 ng/mL)	<3.18 nmol/L (<1 ng/mL) (prepubertal)
Prolactin	185.5 mIU/L (185.5 μIU/mL)	72.66-407.4 mIU/L (72.66-407.4 μIU/mL)
Free T4	12.11 pmol/L (0.94 ng/dL)	9.36-14.49 pmol/L (0.73-1.12 ng/dL)
TSH	1.62 mIU/L (1.62 μIU/mL)	0.35-4.94 mIU/L (0.35-4.94 μIU/mL)
Androstenedione	2 nmol/L (0.58 ng/mL)	0.5-3.4 nmol/L (0.145-0.986 ng/mL)
17-OHP	2.7 nmol/L (0.84 ng/mL)	<6 nmol/L (<1.88 ng/mL)
DHEA-S	1.26 µmol/L (45.88 μg/dL)	0.4-2.2 μmol/L (14.72-80.96 μg/dL)
β-hCG	105 IU/L (105 mIU/mL)	<5 IU/L (<5 mIU/mL)
α-Fetoprotein	<2.25 IU/mL (<272.25 ng/dL)	0-5.8 IU/mL (0-701.8 ng/dL)
LHRH stimulation test		
Peak LH	1.08 IU/L (1.08 mIU/mL)	<5 IU/L (<5 mIU/mL) (prepubertal)
Peak FSH	0.32 IU/L (0.32 mIU/mL)	0-2.8 IU/L (0-2.8 mIU/mL) (prepubertal)
ACTH stimulation test		
Peak cortisol	491 nmol/L (17.8 μg/dL)	>500 nmol/L (>18.1 μg/dL)
Peak 17-OHP	6 nmol/L (1.88 ng/mL)	<30 nmol/L (<9.42 ng/mL)

Values in parenthesis are conventional units.

Abbreviations: 17-OHP, 17-hydroxyprogesterone; ACTH, adrenocorticotropin; β-hCG, β subunit of human chorionic gonadotropin; DHEA-S, dehydroepiandrosterone sulfate; FSH, follicle-stimulating hormone; LH, luteinizing hormone; LHRH, luteinizing hormone–releasing hormone; T4, thyroxine; TSH, thyrotropin.

The increased serum β-hCG levels prompted a comprehensive oncological work-up to rule out a secreting GCT. The chest x-ray and whole-body CT scan were interpreted as normal, including the retroperitoneum and adrenal glands; testicular ultrasound revealed normal pubertal-sized testes; and the brain magnetic resonance imaging (MRI) was unremarkable, except for a 1-cm pineal cyst. The bone scan revealed no evidence of fibrous dysplasia. A lumbar puncture revealed undetectable β-hCG levels, less than 1 mIU/mL (1 IU/L), in the cerebrospinal fluid.

**Figure 1.**
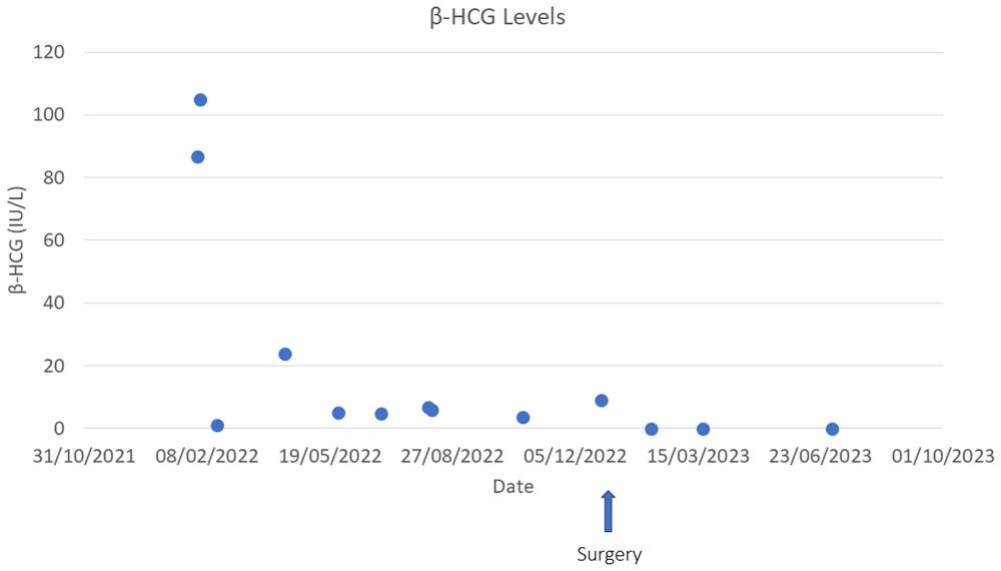
Serum β-human chorionic gonadotropin (β-hCG) levels recorded at different time points.

**Figure 2.**
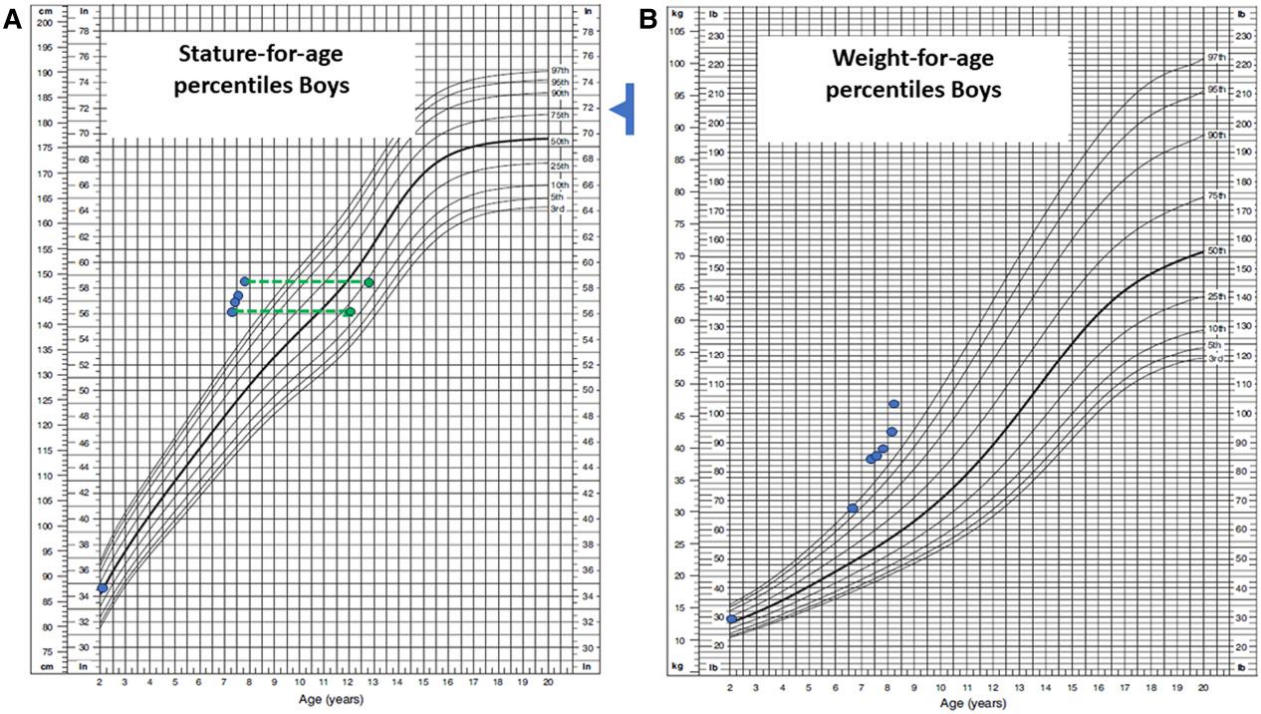
Patient's growth chart according to the Centers for Disease Control and Prevention reference percentiles for A, stature and B, weight. Growth measurements obtained at the visits are presented as blue dots. Green triangles represent the patient's bone age. The blue arrow indicates the familial height target.

Over the subsequent months, the β-hCG levels displayed fluctuations. Approximately 2 weeks following initial presentation, the elevated level of β-hCG decreased to normal range (1 mIU/mL; 1 IU/L). It then rose again to 23.9 mIU/mL (23.9 IU/L) before a reduction toward near-normal levels ranging from 3.6 to 6.9 mIU/mL (3.6 to 6.9 IU/L) (Fig. 1). This led to a spontaneous decrease and increase in testosterone levels from 3.16 to 12.75 nmol/L (90.96 to 367.79 ng/dL). It is worth noting that the patient and his parents consistently denied the use of any drugs or dietary supplements, including diet pills that might contain β-hCG [6]. Clinically, although there was a reduction in testicular volume to 5 mL, there remained a sustained acceleration both in growth rate and bone maturation rate (Fig. 2).

**Figure 3.**
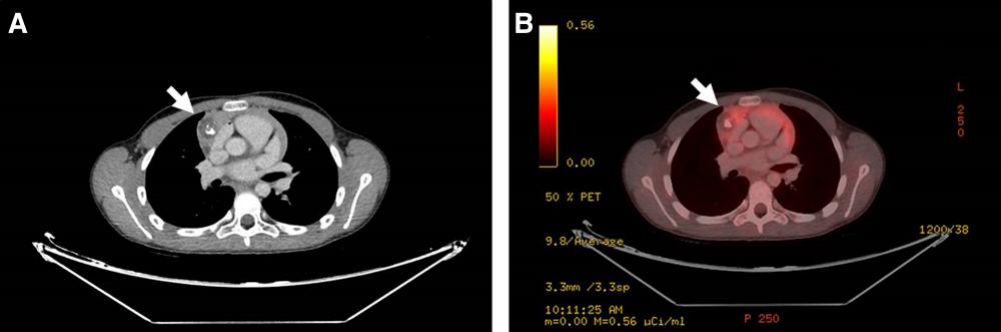
A and B, Fluorodeoxyglucose positron emission tomography–computed tomography (FDG PET-CT) imaging scans showing a hypodense lesion size 37 × 21 × 24 mm with calcifications and macroscopic fat tissue (white arrow). B, In the center of the lesion an increase of F-18 fluorodeoxyglucose (FDG) uptake is shown.

Considering the perplexing course, karyotype testing was performed, ultimately revealing a 47, XXY karyotype, thereby confirming the diagnosis of Klinefelter syndrome (KS). Given the established link between KS and GCTs, our patient underwent a repeated imaging evaluation 10 months after referral to our clinic. The subsequent brain MRI displayed no changes from the initial MRI. However, a FDG PET-CT scan unveiled a mediastinal hypodense lesion measuring 37 × 21 × 24 mm characterized by calcifications and macroscopic fat tissue with increased FDG uptake in the center of the lesion (Fig. 3). In view of the previously elevated β-hCG levels in conjunction with the presence of a mediastinal mass, the clinical diagnosis of a secreting GCT was definite.

## Treatment

The patient underwent tumor resection and subsequent histopathological examination revealed an intrathymic mature cystic teratoma composed of squamous, respiratory type, and mucinous epithelium, cartilage, and bone as well as a well-defined focus of florid vascular proliferation with plump epithelioid endothelial cells, with some morphological resemblance to “hobnail”/glomeruloid hemangioma (positive for CD31 and ERG and negative for TFE3 and C-myc). Following the surgical intervention, β-hCG levels dropped to less than 0.25 mIU/mL (0.25 IU/L).

## Outcome and Follow-up

Six months after the surgical procedure, the young boy reported a state of well-being. The testes had regressed in size to 4 mL bilaterally and were found to be normal on palpation. His pubertal development was categorized as Tanner stage A1 and P3. The pace of linear growth had decelerated to a rate of 5.1 cm per year. Hormone evaluations disclosed serum testosterone levels of 0.79 nmol/L (22.79 ng/dL) (normal prepubertal range <0.24-0.9 nmol/L, <6.92-25.96 ng/dL). Concurrently, the FSH and LH levels were measured at 0.49 mIU/mL (0.49 IU/L) and 0.42 mIU/mL (0.42 IU/L), respectively. Moreover, both β-hCG and α-fetoprotein levels were below the threshold of 0.25 mIU/mL (0.25 IU/L) and 2.25 IU/mL (50.82 ng/dL), respectively.

## Discussion

KS is the most common sex-chromosome aberration in men, but remains underdiagnosed, most likely due to large variation in clinical presentation and insufficient awareness of the syndrome by medical personnel [7]. The most common clinical findings include small testes, azoospermia, hypergonadotropic hypogonadism, learning disabilities, gynecomastia, and cryptorchidism [8]. Boys with KS undergo puberty at the normal time, but testicular growth tends to stop during the intermediate phase of puberty, with the testicles reaching an average volume of 4 to 5 mL [9]. In this case, aside from a slightly slower developmental trajectory in comparison to his siblings (albeit within the typical range), none of the typical indicators for KS were evident. Conversely, the patient presented with an enlarged TV of 8 to 10 mL and penile length reaching 11 cm. The only clinical clue for diagnosing this syndrome was the elevated β-hCG levels.

Notably, while precocious puberty is not typically a manifestation of patients with KS, in the rare subgroup of KS patients with β-hCG–producing tumors, precocious puberty may occur [4, 10]. Furthermore, in these cases, testicular enlargement is not uncommon. A review of boys with KS and GCT who presented with PP found that 4 out of 9 children (44%) had a TV above 3 mL, with 2 of them having a TV of 13 mL [10]. Interestingly, the patient's mother did report eating habits consistent with pica, which was previously reported in this syndrome, among additional eating disorders [11, 12].

Another diagnostic challenge in this case was the interpretation of the fluctuating and relatively low levels of β-hCG (see Fig. 1). At first, the marked decrease in β-hCG levels following the patient's initial presentation led us to consider an exogenous consumption of β-hCG–containing substance at the differential diagnosis. However, the boy and his family denied the consumption of any drugs or food supplements.

Notably, while the initial imaging studies in this case were interpreted as normal, on retrospective review of the initial CT examination, a mediastinal hypodense lesion measuring 8.7 × 11 mm, without calcifications or enhancement, was identified. The lesion was initially considered an artifact due to cardiac pulsations. It is therefore possible that an initial PET-CT would have detected the lesion as it was later characterized by an increased FDG uptake.

In a literature review, we found 2 additional cases of recurrent β-hCG elevation, although in a substantially higher range, in boys presenting with GIPP. In the first case [13], a 9-year-old boy presented with Tanner stage P4, G 4, prepubertal testis volume of 2.5 mL, and highly variable β-hCG levels with peaks of 2353 mIU/mL (2353 IU/L) and repeated spontaneous regressions to normal values. Two years after his presentation, when β-hCG surged up to 286 000 mIU/mL (286 000 IU/L), CT scan examination and an MRI of the chest revealed a tumor mass in the anterior mediastinum measuring 8.5 × 6 cm. Histology revealed choriocarcinoma immature teratoma cells. In the second case [14], a 6.2-year-old boy presented with Tanner pubertal stage of P3 A1, TV of 3 (left) and 4 (right) mL, and penile length of 7.5 cm. Brain imaging detected a bilobed cyst of the pineal gland. Biochemical follow-up revealed a fluctuant trend of β-hCG levels in the range of 0 to 577 mIU/mL (0-577 IU/L). A repeat brain MRI showed further increase in the size of the cyst, and the patient underwent a surgical excision of the lesion. The histology was consistent with a GCT with a predominant mature teratomatous component. Notably in both cases, it was not mentioned whether a karyotype test was performed to rule out KS.

While GCTs produce the intact hCG molecule, most serum assays measure specifically the β-subunit as the α-subunit is identical to the α-subunits of several other hormones, including LH, FSH, and thyrotropin. Its production by the tumor may vary depending on factors such as tumor burden and the histologic subtypes [15]. Factors that might be responsible for the β-hCG fluctuations and to its spontaneous decline in the cases discussed here are unknown. Schwabe et al [13] hypothesized that a specific immunological response was responsible for the undulating β-hCG seen in their case. Another possible hypothesis is that a change in the tumor differentiation into a more differentiated tissue during the follow-up period may have led to the lower hCG secretion. These hypotheses remain to be tested in future studies.

## Learning Points

- Gonadotropin-independent precocious puberty in a boy can be the first clinical sign of a serious underlying disease.
- GCTs in children are rare, but are substantially more prevalent in individuals with KS.
- Although KS is more commonly characterized by hypergonadotropic hypogonadism and small testes (≤4 cc each) in teenage boys, it should also be considered in cases of precocious puberty and enlarged testicular size, if elevated β-hCG levels are detected.
- In boys who exhibits PP and an elevated β-hCG level, repeated imaging scans should be considered even in situations where initial imaging studies are interpreted as normal and β-hCG levels naturally decrease to normal or slightly above normal range.

## Data Availability

Data sharing is not applicable to this article as no data sets were generated or analyzed during the present study.

## Abbreviations

17-OHP: 17-hydroxyprogesterone
β-hCG: β subunit of human chorionic gonadotropin
ACTH: adrenocorticotropin
CPP: central precocious puberty
CT: computed tomography
DHEA-S: dehydroepiandrosterone sulfate
FDG: fluorodeoxyglucose
FSH: follicle-stimulating hormone
GCTs: germ cell tumors
GIPP: gonadotropin-releasing hormone–independent precocious puberty
GnRH: gonadotropin-releasing hormone
KS: Klinefelter syndrome
LH: luteinizing hormone
LHRH: luteinizing hormone–releasing hormone
MRI: magnetic resonance imaging
OR: odds ratio
PET: positron emission tomography
PP: precocious puberty
T4: thyroxine
TSH: thyrotropin
TV: testicular volume
